# An Updated Review of Hypotheses Regarding Bat Attraction to Wind Turbines

**DOI:** 10.3390/ani12030343

**Published:** 2022-01-31

**Authors:** Emma E. Guest, Brittany F. Stamps, Nevin D. Durish, Amanda M. Hale, Cris D. Hein, Brogan P. Morton, Sara P. Weaver, Sarah R. Fritts

**Affiliations:** 1 Department of Biology, Texas State University, 601 University Drive, San Marcos, TX 78666, USA; eeg89@txstate.edu (E.E.G.); bfs27@txstate.edu (B.F.S.); 2 ESE Partners, LLC, 5910 Courtyard Drive Suite 170, Austin, TX 78731, USA; nevin@esepartners.com; 3 Department of Biology, Texas Christian University, Fort Worth, TX 76129, USA; a.hale@tcu.edu; 4 National Renewable Energy Laboratory, Arvada, CO 80007, USA; Cris.Hein@nrel.gov; 5 Wildlife Imaging Systems, 328 Mechanicsville Road, Hinesburg, VT 05461, USA; brogan@wildlifeimagingsystems.com; 6 Bowman, 133 West San Antonio Street #500, San Marcos, TX 78666, USA; sweaver@bowman.com

**Keywords:** attraction, bats, mortality, turbines, wind energy

## Abstract

**Simple Summary:**

The rapid development of wind energy facilities has increased bat mortality due to wind turbine blade strikes. Patterns of bat activity and mortality at wind energy facilities suggest that bats are attracted to wind turbines. It has been more than a decade since a comprehensive review of the various attraction hypotheses was published, highlighting the need to revisit and assess progress in the testing of these ideas. In this review, we discuss the most prominent attraction hypotheses, summarize the current state of knowledge, and briefly outline remaining questions. Identifying the causes of bat interactions with wind turbines is critical to developing effective impact minimization strategies.

**Abstract:**

Patterns of bat activity and mortalities at wind energy facilities suggest that bats are attracted to wind turbines based on bat behavioral responses to wind turbines. For example, current monitoring efforts suggest that bat activity increases post-wind turbine construction, with bats making multiple passes near wind turbines. We separated the attraction hypothesis into five previously proposed explanations of bat interactions at or near wind turbines, including attraction based on noise, roost sites, foraging and water, mating behavior, and lights, and one new hypothesis regarding olfaction, and provide a state of the knowledge in 2022. Our review indicates that future research should prioritize attraction based on social behaviors, such as mating and scent-marking, as this aspect of the attraction hypothesis has many postulates and remains the most unclear. Relatively more data regarding attraction to wind turbines based on lighting and noise emission exist, and these data indicate that these are unlikely attractants. Analyzing attraction at the species-level should be prioritized because of differences in foraging, flight, and social behavior among bat species. Lastly, research assessing bat attraction at various scales, such as the turbine or facility scale, is lacking, which could provide important insights for both wind turbine siting decisions and bat mortality minimization strategies. Identifying the causes of bat interactions with wind turbines is critical for developing effective impact minimization strategies.

## 1. Introduction

Anthropogenic climate change resulting from fossil fuel emissions has sparked interest in renewable energy sources. Wind energy is an appealing source due to low emissions; thus, its global deployment is rapidly expanding [1]. The Global Wind Energy Council reported that total wind energy capacity exceeded 651 gigawatts in 2019, a 19% increase in installation from the previous year [2]. As of 2019, the United States was the second leading market for installed wind energy capacity, only trailing China, together accounting for over 60% of the new capacity in 2019, followed by Germany, India, the United Kingdom, and Spain [2].

Although renewable energy sources are reducing emissions that lead to climate change, wind and other renewables are not without unintended negative impacts [3]. One consequence of wind energy development is bat mortality caused by wind turbine blade strikes, and increasing development of wind energy represents a relatively new stressor to numerous bat species, sparking concern among conservationists and private industries [4]. In North America, migratory tree-roosting species, including the hoary bat (*Lasiurus cinereus*), eastern red bat (*Lasiurus borealis*), and silver-haired bat (*Lasionycteris noctivagans*), constitute most of the bat carcasses reported in the U.S. and Canada from wind turbine strikes, and therefore are thought to be currently the most vulnerable to wind turbine-related mortality in these countries [5,6,7,8,9]. Additionally, wind turbine strikes are known to cause the mortality of protected species, including the Hawaiian hoary bat (*Lasiurus semotus*) [10,11]. Although research regarding bats and wind turbines has occurred to varying degrees worldwide, bat mortality due to wind turbines has been reported across all continents [12], excluding Antarctica, highlighting the potential global effect of wind turbines on bat populations. Understanding whether bat mortality due to wind turbines leads to population-level declines is complicated for most bat species because demographic data and population estimates are difficult to collect and estimate, respectively [13,14,15]. Because of the lack of empirical studies, Frick et al. [16] used expert elicitation to estimate population size and growth rate for the hoary bat in North America, and suggested that under the most likely scenario of a population size of 2.5 million bats and a growth rate of 1.01, hoary bats could decline by 90% in the next 50 years from wind energy impacts alone if minimization strategies are not implemented.

Thermal video observations of bats interacting with wind turbines indicate that some bats may not be randomly colliding with wind turbines, but instead are actively approaching wind turbine components (e.g., tower, nacelle, and blades) and make multiple passes in and around the rotor-swept area [17,18,19]. Additionally, Richardson et al. [20] assessed bat activity using acoustic monitoring and noted greater activity for *Pipistrellus* species at turbine sites compared to control sites with similar habitats, but no differences in the activity of other species in the same genus. Actively flying near wind turbines increases mortality risk, but the underlying behavioral or physiological traits explaining why bats interact with wind turbines remain unknown. In the northern hemisphere, definitive patterns of bat activity and mortality at wind energy facilities have been observed, with peaks occurring during late summer to early autumn (primarily July–September, depending on facility location), and on nights with low, less variable, wind speed conditions [19,21]. This period coincides with the mating season and autumn migration of the aforementioned species [7,22]. The relationship between bat mortality and turbine height or size is less clear, however, with some studies suggesting a positive relationship [23,24,25] and others suggesting a weak [26] or no relationship [27,28]. A recent analysis [29] demonstrated that the relative amount of energy produced, rather than metrics of turbine size, is a better predictor of bat wind turbine-related mortality and warrants further investigation.

Observations of bats interacting with wind turbines, as well as the lack of a predictive relationship between ecological impact assessments or pre-construction acoustic activity with bat mortality during the post-construction phase [30,31], provide increasing evidence that bats may be attracted to wind turbines. Several attraction hypotheses, including attraction based on the noise produced by wind turbines; increased prey availability due to modified landscapes and insect attraction to wind turbines; wind turbines serving as potential roost sites; and wind turbines serving as rendezvous points for mating aggregations have been proposed [13,32,33].

Although the scientific community has made advances in our understanding of why bats are potentially attracted to and killed by wind turbines [13,21,33], there are still many questions that remain. It has been more than a decade since a comprehensive review of the various attraction hypotheses was published [33], highlighting the need to revisit and assess progress in the testing of these ideas. In this review, we discuss the most prominent attraction hypotheses, summarize the current state of knowledge, and outline remaining questions.

## 2. Materials and Methods

We conducted a literature review encompassing studies from peer-reviewed journals, public technical reports, theses/dissertations, and presentations on research from organizations and agencies, comprising 40 studies that either directly or indirectly focused on hypotheses regarding bat attraction to wind turbines. The initial literature was collected from April to July 2020 using web search engines, contacting professionals in the field, and reviewing existing literature citations. Moreover, additional relevant literature was added as it became available in 2021. Search terms including “bats and wind energy”, “bats and wind turbines”, “bat attraction to wind turbines”, “bat attraction hypothesis”, “cause of bat fatalities”, “wind turbine fatalities”, and “patterns of bat fatalities” were used to search web databases made available through the Texas State University Library, including Google Scholar (https://scholar.google.com/, accessed on 1 June 2021), Web of Science (https://clarivate.com/webofsciencegroup/solutions/web-of-science, accessed on 1 June 2021), and Wildlife and Ecology Studies Worldwide (https://www.ebsco.com/products/research-databases/wildlife-ecology-studies-worldwide, accessed on 1 June 2021). 

A comprehensive review of the bat attraction hypothesis was provided by Cryan and Barclay [33], and this review will provide an update to their work. We separated the attraction hypothesis into five previously proposed explanations of bat activity at or near wind turbines to update previous research, including attraction based on noise, roost sites, foraging and water, mating behavior, and light. We also added one new hypothesis regarding olfaction and provided a state of the knowledge as of 2022 for each potential cause of attraction.

## 3. Results and Discussion

**Figure 1:**
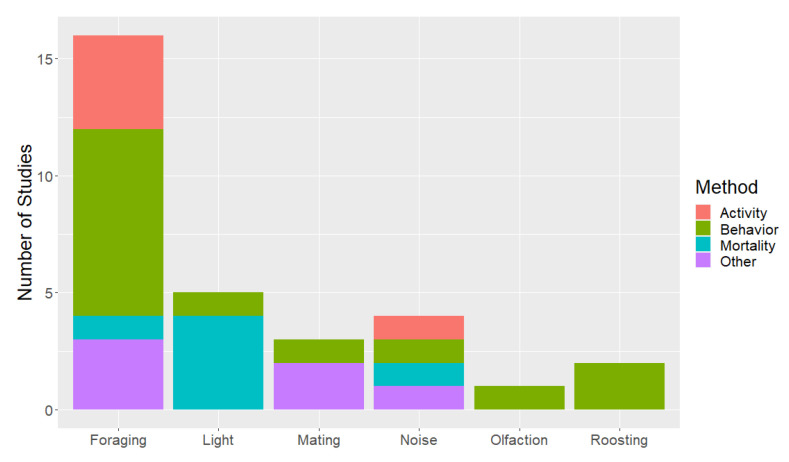
The number of research studies that focused directly on each of the attraction hypotheses separated by the type of response quantified. The number of studies in the table total 31 because some focused on multiple attraction hypotheses and/or used multiple methods.

An overview of research that pertains to bat attraction to wind turbines is in Supplementary Table S1. We determined 25 studies were directly related to bat attraction to wind turbines (Figure 1). In addition, 15 studies were indirectly related to bat attraction to wind turbines. We separated the combined 40 direct and indirect studies by hypothesis, with 23 relating to foraging, 8 to light, 6 to roosting, 5 to noise, and 4 to mating. Several studies include results related to more than one hypothesis. Olfaction was not listed as a potential mechanism for attraction, except in 1 unpublished study. Research questions were assessed through bat mortality (*n* = 5), bat activity (*n* = 13), bat behavior (*n* = 17) and other responses (*n* = 8). The majority of studies focused on work in North America (*n* = 24), followed by Europe (*n* = 15), and worldwide (*n* = 1).

### 3.1. Noise

Bat echolocation calls are among the loudest natural sounds and provide reliable sensory perception for navigating and foraging at night [34]. Evidence of bats using acoustic cues between 3 and 12 kHz from distances of up to several hundred meters prompted the idea that noise emitted from wind turbines could be misinterpreted as prey [34]. Noises emitted by wind turbines potentially attracting bats include sounds created by moving blades, turbine generators or other nacelle electronics, and anemometers [5]. However, several studies have indicated that artificial noise produced by wind turbine nacelles or blades does not propagate far enough from the turbine to attract bats [35,36,37,38]. Szewczak and Arnett [36] concluded that low-frequency ultrasound (20–30 kHz) emitted by wind turbines attenuated at the ground level, and thus the ultrasound is likely indiscernible from ambient sound. Some ultrasonic anemometers on wind turbines emit ultrasound at approximately 38 kHz, a frequency used by species that commonly interact with wind turbines [5]. However, Arnett et al. [5] disabled anemometers at half of the wind turbines at two study sites (*n* = 11, *n* = 5) and observed no significant difference in bat mortality. Another hypothesis proposed that defects in nacelle electronics or blade structure could have potential for emitting noise that could be detected by bats in direct proximity to wind turbines; however, it was determined that noise from defects readily attenuates within 10 m of the wind turbine, making this hypothesis unlikely [36,37]. Furthermore, bats have been observed flying in the direct vicinity and investigating nonmoving wind turbine blades, suggesting an alternate or additional explanation aside from noise, assuming that nonmoving blades produce no sound [5,17,35]. In conclusion, whereas bats have been shown to use natural sounds in the landscape to navigate to potential foraging grounds, previous research has shown that possible sources of ultrasonic noise from turbine structure attenuate over relatively short distances [5,36,37], suggesting that bat attraction to wind turbine-generated ultrasound is unlikely.

### 3.2. Roosting

It is hypothesized that the tall, stand-alone structure of wind turbines on the landscape could be misinterpreted by bats as trees and viewed as potential roost structures [33]. Bat attraction to tall structures may be attributed to bat behavior to select trees with favorable roosting habitat characteristics [13,33,39]. Tree height is an important characteristic for roost selection, with taller, larger trees favored by several species [40,41]. Using thermal imaging, bats have been observed investigating both moving and nonmoving wind turbine blades and towers, suggesting attraction to these stand-alone structures for potential roosts [5,17], although investigatory behaviors of stand-alone structures could be indicative of other hypotheses regarding bat attraction to wind turbines. If swarming signals are used to ensure group cohesion at wind energy facilities as they are at other roost sites, the influx in bats around wind turbines due to this behavior could be contributing to increased mortality [42]. Another indication that bats may use turbines as roosts, either night or day roosts, is the presence of guano from several bat species at searchable locations on wind turbine towers, transformers, and doors [43], which was further supported by night vision surveys in which bats were observed entering or exiting these wind turbine structures at night [44]. Further research is needed to determine the extent to which roosting behavior could be attracting bats to wind turbines. 

### 3.3. Foraging and Water

Insect density around wind turbines may be positively correlated with bat activity, because bats may perceive wind turbine sites as a potential food source. The proposed logic for the accumulation of insects near wind turbines includes hilltopping behavior, insect attraction to the light or heat emitted from wind turbine structures, and insect attraction to wind turbine color [35,45,46]. Hilltopping behavior involves the congregation of insects at the highest point in the local landscape to improve the likelihood of mating success and may, in part, explain increased levels of bat mortality due to wind turbines located on hilltops and ridges [21,46,47]. However, research relating hilltopping behavior to nocturnal moths commonly eaten by insectivorous bat species is lacking [48,49,50]. Wind turbine structures are typically white or light grey in color, which has been demonstrated to be significantly more attractive to insects during the day and one hour after sunset compared to other colors [45], furthering the potential for bats to be attracted to wind turbines because of increased prey availability. Additionally, insect swarming at the top of wind turbine structures has been observed using lidar technology, with insect swarms dispersing just after sunset [51]. Nonetheless, high insect abundance around wind turbine structures may provide an opportunity for bats to associate wind turbines with quality foraging habitats.

The potential use of wind turbine sites as a food source by bats likely varies by species and is largely determined by insect foraging habits and the composition of the local insect community. Migration patterns of Brazilian free-tailed bats coincide with concentrations of migratory moths, a known and important food source for this species [52,53,54]. For most bat species, there is a paucity of information regarding migratory movements and foraging habits, but it is believed that migratory bats use stopover sites to feed during migration routes rather than storing an abundance of fat. Some evidence of foraging activity along migratory routes has been observed through tracking the movement of radio-tagged individuals [55,56,57], stable isotope analysis [58], and direct observation [59]. This may explain greater rates of mortality due to wind turbines for migratory compared to non-migratory bat species [5,8,60,61].

Bats exhibit foraging flight behavior in the vicinity of wind turbines and among wind turbine blades [5,17,35]. The most common method to assess foraging behaviors around wind turbines is using acoustic detectors, but acoustic activity has caveats. Acoustic monitoring has been used to assess the occurrence of foraging or approach-phase echolocation calls as well as feeding buzzes to evaluate bat activity and behavior at wind turbine and tower structures [38,62,63,64,65]. All six bat species known to occur at a study site in north-central Texas, United States, were recorded using feeding buzzes at nacelle height (*n* = 63) and at ground level (*n* = 50), although feeding buzzes were recorded in only 3.1% of total bat passes recorded, indicating bats likely were not foraging near the rotor-swept area [63], a conclusion that potentially would have been overlooked if all bat passes were included in the analysis. Additionally, the acoustic characteristics of a feeding buzz are similar to the acoustic traits emitted by bats attempting to land on an object [66] or approaching water [67], so the overestimation or misclassification of feeding buzzes is possible [63]. At a site in Alberta, Canada, the number of feeding buzzes was greater at meteorological towers than wind turbine structures, suggesting that insects may congregate at other tall structures [65]. The variation among studies indicates that regional differences in species and foraging behavior may exist. Overall, most attempts to assess bat activity at wind turbines via acoustic detectors resulted in little to no evidence of foraging or feeding behavior being a primary cause of bat activity at wind turbines, as feeding buzzes were not consistently detected in the rotor-swept area [38,62,64,65]. However, other methodologies including assessing bat stomach contents and behavior sometimes contradict this conclusion. The inability of ultrasonic microphones to capture the entire rotor-swept zone must be considered, as the possibility of missed bat passes exists when using detectors in this capacity [68,69]. Conversely, Horn et al. [17] used thermal imaging to observe bat activity at wind turbines and concluded insect passes were a predictor of bat activity, promoting the correlation between weather variables and insect seasonality to the cyclical timing of bat mortality at wind turbines. 

An analysis of stomach fullness and stomach contents from bat carcasses collected around wind turbines [50,63,70], as well as genetic analysis of feces from bat carcasses found around wind turbines [63], provides additional means of assessing the likelihood of the attraction hypothesis of wind turbines as a foraging resource. Insect remains frequently occur in the stomachs of known migratory species found dead around wind turbines, supporting the idea that bats feed during migration [63,71]. Foo et al. [63] analyzed all three of the aforementioned sample sources for eastern red bats and hoary bats (*n* = 45, *n* = 23, respectively), two species commonly found dead at wind turbines. In two consecutive years between the months of July and August, the authors found that eastern red bat and hoary bat carcasses had stomachs that were full or partially full (*n* = 20, *n* = 15, respectively), and common insects encountered in stomach content analysis were observed at wind turbines during insect surveys, indicating that bats were potentially feeding at wind turbines prior to death [63]. Additionally, stomach contents analysis of bat carcasses found under wind turbines provided evidence of bats eating nonflying insects presumably found resting on wind turbine structures [50], which may indicate that some bat species capture prey from the surface of wind turbines [63]. In contrast, Valdez et al. [70] examined 57 hoary bat carcasses at wind turbines located in western New York (*n* = 4) and central Texas (*n* = 1) between July and September and noted a lack of insects in the mouths and esophagi of all individuals involved in the study, suggesting that bats were not in the process of feeding at the exact time of death. However, even if bats were foraging immediately before wind turbine-related mortality, the impact of collision may influence whether prey would remain present in the mouths and esophagi of bat carcasses.

Additional motives for increased foraging activity at wind turbine structures resulting in mortality have been proposed. First, it has been found that bats perceive acoustically smooth surfaces, such as metal or plastic, to be water—despite conflicting information from other sensory mechanisms [72,73]. This is perhaps due to the similar acoustic qualities of these materials regarding the reflection of bat echolocation off these surfaces [44,73]. Research suggests that the echolocation characteristics reflected from water surfaces could cause the smooth surface of wind turbines to be misinterpreted as water [44,72,73]. Second, hypotheses relating landscape features of wind energy facilities to increased bat activity through indirect attraction for foraging have been made, but tests of this relationship are lacking [5,33]. Several bat species exhibit preferences for foraging and commuting along linear landscapes, forest edges, and forest gaps [74,75]. These habitat features are commonly created when roads and wind turbine pads are built during construction and may indirectly cause increased bat activity around wind turbines, resulting in mortality [13].

### 3.4. Mating

It is suggested that male and female bats of some species visually orient toward the tallest trees on the landscape for navigation towards mating aggregations [39]. Direct observation through thermal imaging coupled with mortality trends suggest that wind turbines may be used by bats for orientation to potential mating sites [17,32,39]. Knowledge of mating strategies for a large proportion of bat species is incomplete, but observations of tree bat behavior in Europe indicate that similar species may use lekking and resource-defense behaviors to attract females [39]. These mating systems lead to aggregations of individuals and align with increases in mortalities around wind turbines during the autumn season, as well as the higher rate of carcasses presumably being adults compared to juveniles [5,24,32,76,77], although possible age and sex misclassification [78,79,80] and males and females of some species potentially mating during their first autumn [81] must be considered when evaluating patterns from mortality studies. Furthering this idea, Cryan et al. [81] observed spermatozoa in the epididymis of 89% of hoary (*n* = 70), 100% of eastern red (*n* = 15), and 67% of silver-haired (*n* = 6) adult male bat carcasses collected under wind turbines from July to October, suggesting male readiness to mate. Additionally, acoustic recordings have shown observations of multiple individuals simultaneously at single wind turbine towers with only a small proportion of feeding buzzes [62], as well as thermal recordings of bats flying in pairs, although in small proportion to overall observations [18], offering mating aggregation behavior as a potential explanation. However, visual identification of the call type is difficult, and ultrasonic social calls are not well-characterized for many North American bat species and could be misidentified, and therefore underrepresented in acoustic analyses of bat behavior at wind turbines [33]. Conversely, Brazilian free-tailed bats, a species that accounts for a large percentage of carcasses found beneath wind turbines located within their geographic range [21,82], do not mate during late summer and early autumn, when peak mortalities at wind turbines occur [83], highlighting variation in the potential causes of bat attraction to wind turbines across species. Support for bat attraction to wind turbines based on mating is primarily related to the overlap in the timing of peak mortalities and mating season, and although attraction to wind turbines for mating opportunities may occur, the hypothesis lacks substantial research to provide evidence of a relationship.

### 3.5. Lights

Lights on wind turbines or associated infrastructure (e.g., operations and maintenance facilities) may directly or indirectly contribute to increased bat activity. For example, bats may orient towards light of certain wavelengths during migration or be attracted by insect concentrations near illuminated areas [32,64,84]. The influence of artificial light on bats is species-specific and often based on the species’ morphology [85]. In the United Kingdom, a study suggested that LED streetlights disrupt commuting routes and reduce bat activity for slow-flying species, such as the lesser horseshoe bat (*Rhinolophus hipposideros*) and *Myotis* species [86]. The authors suggested that lights increased risk of predation and therefore potentially restricted bat movement to foraging grounds [86]. In contrast [87,88], other research reported increased foraging activity near flood lights for Kuhl’s pipistrelle bats (*Pipistrellus kuhlii*), a species that occurs throughout southern Europe, north Africa, and west Asia, suggesting the perception of light wavelength and/or foraging behavior as potential indicators for light’s effect on bat species. Using acoustic monitoring in North America, Seewagen et al. [89] concluded that eastern red and hoary bats displayed no significant differences in activity between dark and LED-lit conditions.

Various light colors and wavelengths have differing effects on bat activity, and the influence is also species-specific [85] and may vary with location and season. For example, the medium wavelength of green light has opposing effects depending on species. Voigt et al. [64] observed a 50% increase in acoustic activity of European migratory bat species during green-light treatments compared to darkness, attributing the attraction to positive phototaxis instead of attraction to insect activity due to a similar number of feeding buzzes between treatment and control periods. Furthermore, bat species with opportunistic feeding behavior demonstrated increased activity around treatment posts with green lighting, whereas other species characterized behaviorally as slow flyers avoided the treatment posts, furthering the hypothesis that differences in foraging behavior could contribute to the species-dependent nature of light attraction [90].

In a study investigating the effect of red light, Voigt et al. [84] observed an increase in activity for a single European migratory species, soprano pipistrelle bats (*Pipistrellus pygmaeus*), at red-light treatment poles, but did not document an increase in feeding activity. Variation among species in response to red light could be explained in part by differences in migratory behavior. Migratory bats, such as the soprano pipistrelle, may have increased susceptibility to light attraction because of an increased dependence on vision for navigation and orientation instead of echolocation during long-distance flight [32,84]. Additionally, it is suggested that increased foraging activity due to increased insect activity is not linked to the presence of red light, as insects are attracted to short wavelengths compared to long wavelengths [84]. Conversely, several studies, most of which were conducted at wind energy facilities, reported no relationship between bat activity or mortality with the presence or absence of red light for some bat species [5,28,76,90,91]. For example, at a wind energy facility in Texas, United States, mortality monitoring surveys reported greater mortality around wind turbines without flashing red aviation lights compared to wind turbines with flashing red aviation lights [91]. However, this significant difference was driven by a single species, eastern red bats, with no other significant differences in mortality for other species [91]. Fiedler et al. [28] used mortality monitoring surveys at a wind energy facility in Tennessee, United States, a location within the range of eastern red bats, and reported no significant differences between lit and unlit wind turbines for this species, or any other species. Whereas the overall effect of artificial light on bats has demonstrated variable responses across numerous species, in regard to research conducted at wind energy facilities, artificial lights do not appear to be the primary cause of bat attraction to wind turbines.

### 3.6. Olfaction

Recent work by Tyler et al. [92] using paired thermal video and acoustic recording at meteorological tower sites in south Texas documented “swarming” activity concentrated at various focal points on the meteorological tower structure, including mechanical anemometers, wind vanes, the top of the tower, and aircraft marker balls. The behaviors around the focal points were notable due to bats appearing to make contact, often multiple times, with the structure, particularly when more than one bat was in the area. These observations led to the hypothesis that bats are engaging in scent-marking of focal areas on the tower structure. Scent-marking behavior and its role in social communication in bats were reviewed by Dechmann and Safi [93] and Chaverri et al. [94]. The latter study hypothesized a role for scent-marking associated with territoriality of bat species; however, there are currently no studies of this behavior outside of bat roosts. Scent-marking of tall structures on the landscape by bats would help explain behaviors observed via thermal video at wind turbine structures as well (e.g., [44,95]). Bats approaching wind turbine structures from the leeward side may be an indication of scent-seeking behavior, although this may also suggest foraging behavior, as flying insects accumulate on the leeward side of windbreaks [96,97]. Additionally, videos of repeated visits to, and contact with, a particular portion of the wind turbine structure may suggest bat attraction to specific locations on the wind turbine that had previously been marked by other individuals.

## 4. Conclusions

The available data suggest that several species of bats may be attracted to wind energy facilities or wind turbines, but the cause(s) and scale(s) remain unknown. The attractant(s) may be species-specific and may not be mutually exclusive. Moreover, the habitat conditions within and surrounding wind energy facilities may influence how bats respond to wind turbines. The physiological and behavioral traits associated with attraction must first be identified and understood to minimize bat activity at wind turbines. Whereas some potential sources for bat attraction to wind turbines have been investigated to a relatively greater extent, other explanations remain ill-defined and largely untested. An increase in research regarding bat behavior and flight altitude during migration using GPS tags is warranted to understand how and why bats are moving through wind energy facilities. However, with specific regard to bat attraction, research should be prioritized toward attraction based on social behaviors, such as mating, as this aspect of the attraction hypothesis has many postulates and remains the most unclear. Attraction to wind turbines based on lighting and noise emissions appears unlikely given the available data. Experimental designs should consider the potential difficulty in discriminating between each behavior associated with bat attraction to wind turbines. Additionally, species identification is not discernable with methodologies such as direct observation of flying bats or thermal/near infrared cameras. While these methods of monitoring are aiding in our knowledge of bat behavior and activity at wind energy facilities, species identification should be accounted for in analysis, or analyses should test for species-specific effects related to differences in foraging, flight, and social behaviors that exist among species. Future research should refrain from pooling species for analysis, as this may lead to overgeneralizations occurring due to differences in species behavior. Causes of wind turbine-related mortalities are likely multifaceted and encompass physiological and behavioral aspects in concert with habitat variables and weather, such as wind speed and temperature. Identifying the cause of bat interaction with wind turbines is critical to developing optimal impact minimization strategies. It is important to recognize that research conducted in specific regions may limit broad interpretation elsewhere. We encourage hypothesis testing in other regions to advance our understanding of bat attraction hypotheses.

## Data Availability

Not applicable.

## Supplementary Materials

The following supporting information can be downloaded at: https://www.mdpi.com/article/10.3390/ani12030343/s1, Table S1: Summary of studies directly or indirectly relating to bat attraction to wind turbines.
